# Changing Patterns in Epidemiology of Malaria Between 2006 and 2018 in the South of Fars Province, Southern Iran: The Fall and Rise of Malaria

**DOI:** 10.5334/aogh.2850

**Published:** 2020-07-10

**Authors:** Hamed Delam, Nasrin Shokrpour, Hossein-Ali Nikbakht, Soheil Hassanipour, Khalil Safari, Mohammad-Rafi Bazrafshan

**Affiliations:** 1 Student Research Committee, Larestan University of Medical Sciences, Larestan, IR; 2 English Department, Shiraz University of Medical Sciences, Shiraz, IR; 3 Social Determinants of Health Research Center, Health Research Institute, Babol University of Medical Sciences, Babol, IR; 4 Gastrointestinal and Liver Diseases Research Center, Guilan University of Medical Sciences, Rasht, IR; 5 Department of Medical Entomology, School of Health, Shiraz University of Medical Sciences, Shiraz, IR; 6 Department of Nursing, School of Nursing, Larestan University of Medical Sciences, Larestan, IR

## Abstract

**Background:**

Malaria is one of the major human health problems that have become increasingly important in recent decades.

**Objective:**

The present study aimed to identify the epidemiological status of malaria in the years 2006–2018 in the southern region of Fars province in southern Iran.

**Methods:**

This is a cross-sectional descriptive-analytical study. The study population consisted of all persons with malaria referred to the Center for Disease Control in the four cities of Larestan, Gerash, Evaz and Khonj in the south of Fars province, Southern Iran, between 2006 and 2018. Frequency (%) was used to report descriptive statistics and mean and standard deviation for quantitative variables. The trend of malaria incidence during these years was analyzed using the Cochran Armitage Test. The significance level was considered 5%.

**Findings:**

A total of 190 cases of malaria in the period of 2006 to 2018 occurred in the southern region of Fars province; 77.9% were men, more than 95% were Afghans, and most of them were workers. The incidence of malaria in one hundred thousand people per month showed that most of the new malaria cases were in the months of July to October. The peak incidence was in August, with 19.88 cases per 100,000 people. Cochran-Armitage test results showed that this trend was not statistically significant (P = 0.399), despite an almost upward trend in malaria incidence in the south of Fars province.

**Conclusion:**

The results of this study showed that the trend of malaria in the south of Fars province was ascending; therefore, increasing surveillance activities to prevent and control malaria in such area is of utmost importance.

## Introduction

Malaria is one of the major human health problems that have become increasingly important in recent decades [1]. The disease is transmitted to the human body by the *Anopheles* mosquito infected with *Plasmodium* parasite [2]. There are several species of *Plasmodium* parasites that can infect humans, but the most common ones are *Plasmodium falciparum, P. vivax, P. malariae*, and *P. ovale* [3]. The deadliest and most severe form of malaria worldwide is caused by *Plasmodium falciparum* although other species of *Plasmodium* can also cause severe human disease [4]. There is evidence today that *P. vivax* should not be considered benign because it has been reported to cause life-threatening symptoms, especially in children and pregnant women [5]. This species is common in tropical and subtropical regions, especially Asia and Latin America [6,7]. Since female *Anopheles* mosquitoes need high-grade water and climates to grow and reproduce, malaria is more commonly found in warmer regions closer to the equator, especially in tropical and subtropical countries [8]. More than 90% of malaria cases are found in Africa, 7% in Southeast Asia, and 2% in the Eastern Mediterranean region [1]. According to the World Health Organization report, about 300–500 million cases of malaria are reported annually worldwide, with approximately one million of them causing death; these deaths occur especially in developing countries [3]. According to the World Malaria Report in 2018, about 219 million malaria cases occur annually, of which 435,000 deaths are reported [9]. Most of the deaths were related to young children and other high-risk groups, including pregnant women, insecure travelers, refugees, displaced persons, and workers who had entered endemic areas [10]. Malaria-related deaths and illnesses have a significant impact on the economy of developing countries. Many countries with high numbers of malaria are underdeveloped countries, and the presence of malaria results in the maintenance of disease cycles, poverty, and low levels of health in the community [11]. In 2015, malaria was endemic in 91 countries, most of which were in southern regions of the African deserts. In these areas, more than 90% of malaria cases and 92% of malaria deaths occur [12]. The World Health Organization estimates that between 2000 and 2015, there has been a 41% decrease in the number of new malaria cases worldwide [12], but no significant decrease in the number of cases from 2015 to 2017 [13]. The number of malaria cases in Iran in 2002 was estimated at 10,000, most of which occurred in the summer. The disease is endemic in southeastern Iran, and according to a 2008 report by the Iranian Ministry of Health and Medical Education, about 8% of malaria cases were caused by *P. falciparum* and 90% by *P. vivax* [14]. Although more than 50 years have passed since the program of malaria control interventions in Iran, the disease remains a major health concern in Iran, especially in the south and southeast [15]. Iran is expanding the local transmission of the disease due to its proximity to endemic countries such as Afghanistan and Pakistan and the influx of travelers and migrants from these countries to Iran [15]. Due to the prevalence of malaria in most areas of southern and southeastern Iran – especially in the southern regions of Fars province – as well as the lack of new studies and research in the field of malaria epidemiology in such locations, it is important to understand the epidemiology and discovery of new cases of malaria in Iran. This study was designed to determine the number of new cases and the epidemiological status of malaria in southern Iran between 2006 and 2018.

## Methods

### Study type

This is a cross-sectional descriptive-analytical study.

### Study area

**Figure 1:**
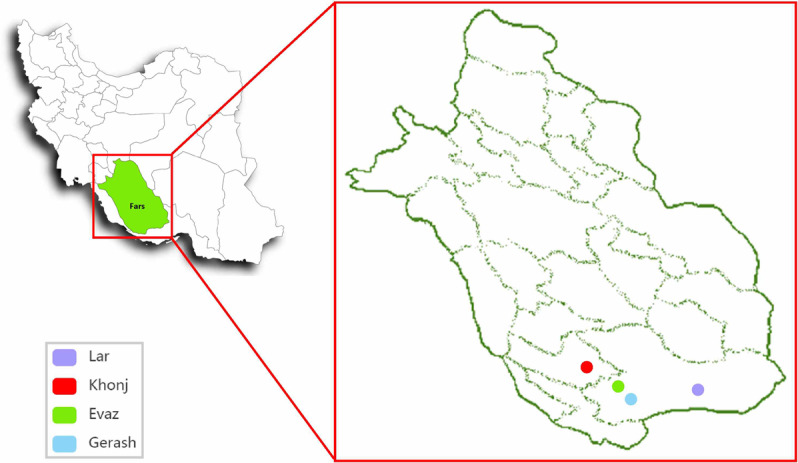
Geographical location of the study.

The study population consisted of all individuals with malaria who were referred to the Center for Disease Control in the four cities of Larestan, Gerash, Evaz and Khonj in southern Fars province, southern Iran, between 2006 and 2018. Larestan is located in the south of Fars province and is one of the largest areas of this province (Figure 1).

### Ethics Committee approval

The present study was the result of research project No. 006-1398 and Code of Ethics IR.LARUMS.REC.1398.014 approved by Larestan University of Medical Sciences.

### Checklist

To collect the data, a checklist was used that includes information such as: age, sex, place of residence, occupation, year and month of occurrence, *Plasmodium* species, previous disease history, family history of disease, type of care, and travel to endemic areas of malaria. The researchers investigated all records of malaria cases in the Center for Communicable Diseases of Larestan between 2006 and 2018 from daily visits. This center records data pertaining to four cities in the south of Fars province: Larestan, Gerash, Evaz, and Khonj. After completing the checklists, the data were entered into SPSS, version 25. During all stages (completing a checklist and entering data into the software), patient information was kept confidential.

### Statistical analysis

Descriptive statistics of the variables are represented by tables and graphs. Frequency (percentage) was used to measure qualitative variables while mean and standard deviation were used for quantitative variables. The trend of malaria incidence during these years was analyzed using the Cochran-Armitage trend test. The significance level was considered 5%.

## Results

**Figure 2:**
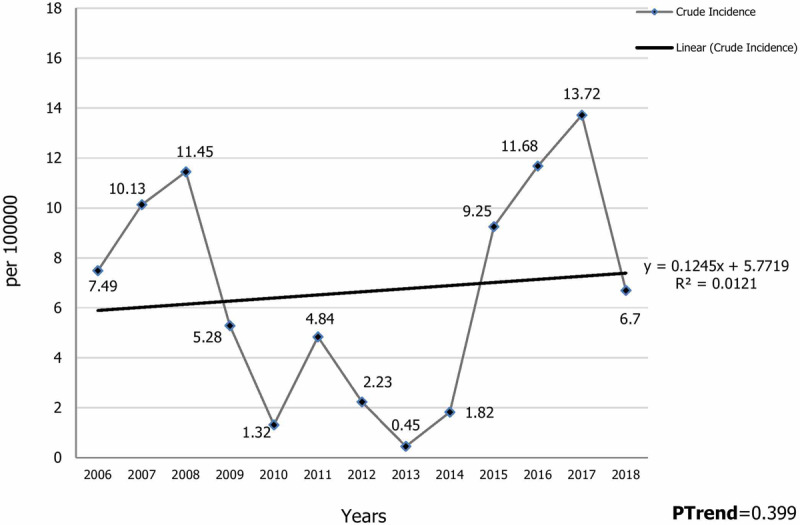
Time trend of Malaria incidence by year per 100,000 persons in Southern Fars Province, (2006–2018).

**Figure 3:**
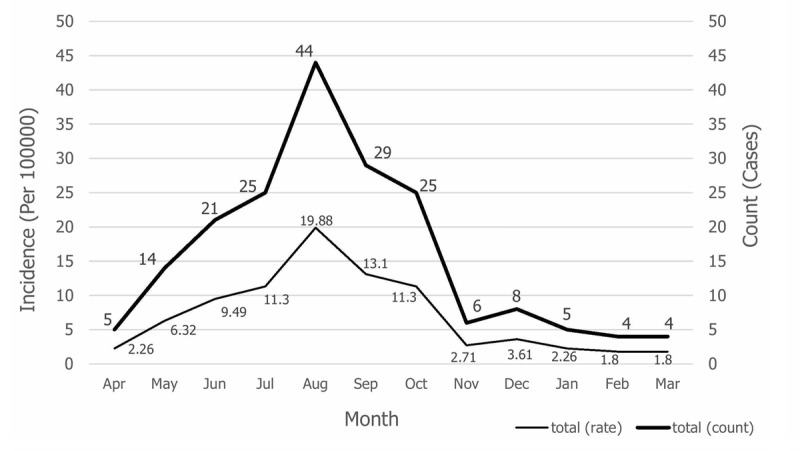
Frequency and incidence of malaria per 100,000 persons per month, south of Fars province, (2006–2018).

**Figure 4:**
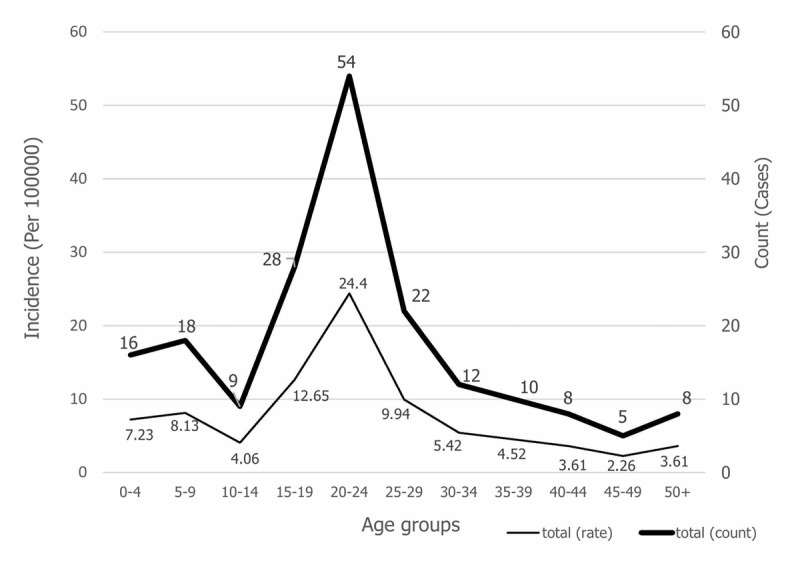
Frequency trend and incidence of Malaria in 100,000 people in 5-year age groups in Southern Fars Province, (2006–2018).

Of the 190 cases of malaria that occurred between 2006 and 2018 in the south of Fars province, 77.9% were men, more than 95% were Afghans, and most were workers (Table 1). During this time period, males were more affected than females; the major *Plasmodium* in this region was *P. vivax*, and from 2016 to 2018 all cases of *P. vivax* were reported. In terms of epidemiological classification, the majority of cases were introduced. Interestingly, no indigenous cases have occurred in this area. Except for the year 2014, the majority of people had a previous history of malaria (Table 1). The Cochran Armitage Trial test was used to analyze the time course of malaria cases per year in the region per 100,000 people. Results showed that this trend was not statistically significant (P = 0.399), despite a slightly upward trend of malaria incidence in southern Fars province. The highest incidence was in 2017, 2016, and 2008, respectively, and the lowest was in 2013 (Figure 2). The frequency trend and incidence of malaria in one hundred thousand people per month showed that most of the new malaria cases occurred in the months from July to October. The peak incidence was in August, with 19.88 cases per 100,000 persons and the lowest incidence was in winter (Figure 3). Also, the frequency trend and incidence of malaria per 100,000 people by age group showed that the highest number of new cases was in the age group of 20–24 years, while the lowest incidence was in the 45–49 year age group (Figure 4). The figure showed that the incidence of malaria in the 0–4 year age group was 7.23 per 100,000 people (Figure 4).

**Table 1:** Comparison of the frequency (%) of qualitative characteristics of people with Malaria by the year of occurrence.

Variables	Total	Year of occurrence												

		2006	2007	2008	2009	2010	2011	2012	2013	2014	2015	2016	2017	2018

**Gender**														
Male	148 (77.9)	15 (88.2)	21 (91.3)	17 (65.4)	9 (75.0)	3 (100)	11 (100)	3 (60.0)	1 (100)	4 (100)	12 (60.0)	17 (68.0)	23 (79.3)	12 (85.7)
Female	42 (22.1)	2 (11.8)	2 (8.7)	9 (34.6)	3 (25.0)	0 (0.0)	0 (0.0)	2 (40.0)	0 (0.0)	0 (0.0)	8 (40.0)	8 (32.0)	6 (20.7)	2 (14.3)
**Nationality**														
Iranian	6 (3.2)	1 (5.9)	1 (4.3)	1 (3.8)	1 (8.3)	0 (0.0)	0 (0.0)	0 (0.0)	0 (0.0)	0 (0.0)	2 (10.0)	0 (0.0)	0 (0.0)	0 (0.0)
Afghan	182 (95.7)	16 (94.1)	22 (95.7)	25 (96.2)	11 (91.7)	3 (100)	9 (81.8)	5 (100)	1 (100)	4 (100)	18 (90.0)	25 (100)	29 (100)	14 (100)
Pakistani	2 (1.1)	0 (0.0)	0 (0.0)	0 (0.0)	0 (0.0)	0 (0.0)	2 (18.2)	0 (0.0)	0 (0.0)	0 (0.0)	0 (0.0)	0 (0.0)	0 (0.0)	0 (0.0)
**Job**														
Housewife	18 (9.5)	2 (11.8)	1 (4.3)	2 (7.7)	2 (16.7)	0 (0.0)	0 (0.0)	0 (0.0)	0 (0.0)	0 (0.0)	5 (25.0)	2 (8.0)	3 (10.3)	1 (7.1)
Child	33 (17.4)	0 (0.0)	5 (21.7)	8 (30.7)	2 (16.7)	0 (0.0)	0 (0.0)	0 (0.0)	0 (0.0)	0 (0.0)	3 (15.0)	9 (36.0)	5 (17.2)	1 (7.1)
Student	8 (4.2)	1 (5.9)	2 (8.7)	0 (0.0)	0 (0.0)	0 (0.0)	0 (0.0)	2 (40.0)	0 (0.0)	0 (0.0)	0 (0.0)	2 (8.0)	0 (0.0)	1 (7.1)
Worker	124 (65.2)	13 (76.4)	15 (65.3)	12 (46.2)	7 (58.3)	3 (100)	11 (100)	3 (60.0)	1 (100)	3 (75.0)	12 (60.0)	12 (48.0)	21 (72.5)	11 (78.7)
Other	7 (3.7)	1 (5.9)	0 (0.0)	4 (15.4)	1 (8.3)	0 (0.0)	0 (0.0)	0 (0.0)	0 (0.0)	1 (25.0)	0 (0.0)	0 (0.0)	0 (0.0)	0 (0.0)
**Residence**														
City	145 (76.3)	15 (88.2)	19 (82.6)	13 (50.0)	7 (58.3)	3 (100)	8 (72.7)	1 (20.0)	1 (100)	2 (50.0)	14 (70.0)	23 (92.0)	26 (89.7)	13 (92.9)
Village	45 (23.7)	2 (11.8)	4 (17.4)	13 (50.0)	5 (41.7)	0 (0.0)	3 (27.3)	4 (80.0)	0 (0.0)	2 (50.0)	6 (30.0)	2 (8.0)	3 (10.3)	1 (7.1)
***Plasmodium* species**														
*Vivax*	163 (85.8)	13 (76.4)	16 (69.6)	18 (69.2)	9 (75.0)	3 (100)	10 (90.9)	4 (80.0)	1 (100)	4 (100)	18 (90.0)	24 (96.0)	29 (100)	14 (100)
*Falciparum*	8 (4.2)	2 (11.8)	1 (4.3)	3 (11.5)	0 (0.0)	0 (0.0)	0 (0.0)	1 (20.0)	0 (0.0)	0 (0.0)	1 (5.0)	0 (0.0)	0 (0.0)	0 (0.0)
Mixed*	19 (10.0)	2 (11.8)	6 (26.1)	5 (19.2)	3 (25.0)	0 (0.0)	1 (9.1)	0 (0.0)	0 (0.0)	0 (0.0)	1 (5.0)	1 (4.0)	0 (0.0)	0 (0.0)
**Past history**														
Has	123 (64.7)	14 (82.4)	15 (65.2)	14 (53.8)	9 (75.0)	2 (66.7)	9 (81.8)	3 (60.0)	1 (100)	1 (25.0)	14 (70.0)	14 (56.0)	19 (65.5)	10 (71.4)
Hasn’t	67 (35.3)	3 (17.6)	8 (34.8)	12 (46.2)	3 (25.0)	1 (33.3)	2 (18.2)	2 (40.0)	0 (0.0)	3 (75.0)	6 (30.0)	11 (44.0)	10 (34.5)	4 (28.6)
**Family history**														
Has	29 (15.3)	0 (0.0)	4 (17.4)	9 (34.6)	2 (16.7)	0 (0.0)	1 (9.1)	0 (0.0)	0 (0.0)	0 (0.0)	2 (10.0)	6 (24.0)	5 (17.2)	0 (0.0)
Hasn’t	161 (84.7)	17 (100)	19 (82.6)	17 (65.4)	10 (83.3)	3 (100)	10 (89.9)	5 (100)	1 (100)	4 (100)	18 (90.0)	19 (76.0)	24 (82.8)	14 (100)
**Surveillance**														
Active	31 (16.3)	2 (11.8)	3 (13.0)	8 (30.8)	1 (8.3)	0 (0.0)	3 (27.3)	0 (0.0)	0 (0.0)	0 (0.0)	1 (5.0)	5 (20.0)	8 (27.6)	0 (0.0)
Inactive	159 (83.7)	15 (88.2)	20 (87.0)	18 (69.2)	11 (91.7)	3 (100)	8 (72.7)	5 (100)	1 (100)	4 (100)	19 (95.0)	20 (80.0)	21 (72.4)	14 (100)
**Epidemiologic class**														
Introduced	160 (84.2)	13 (76.4)	17 (73.9)	19 (73.1)	8 (66.7)	1 (33.3)	8 (72.7)	5 (100)	1 (100)	2 (50.0)	20 (100)	25 (100)	27 (93.1)	14 (100)
Relapse	30 (15.8)	4 (23.6)	6 (26.1)	7 (26.9)	4 (33.3)	2 (66.6)	3 (27.3)	0 (0.0)	0 (0.0)	2 (50.0)	0 (0.0)	0 (0.0)	2 (6.9)	0 (0.0)
**Travel to endemic areas**														
Has	116 (61.1)	8 (47.1)	15 (65.2)	20 (76.9)	5 (41.7)	1 (33.3)	5 (45.5)	5 (100)	1 (100)	4 (100)	11 (55.0)	16 (64.0)	13 (44.8)	12 (85.7)
Hasn’t	74 (38.9)	9 (52.9)	8 (34.8)	6 (23.1)	7 (58.3)	2 (66.6)	6 (54.5)	0 (0.0)	0 (0.0)	0 (0.0)	9 (45.0)	9 (36.0)	16 (55.2)	2 (14.3)

* P. vivax + P. falciparum.

## Discussion

The present study examined malaria data over a 13-year period to identify the epidemiological status of this disease in southern Iran. The study found that between 2006 and 2018, 190 cases of malaria were detected in the south of Fars province in Southern Iran. Men were more affected than women in every year. In the years 2010, 2011, 2013 and 2014, all malaria cases were men. A study by Vatandoost et al. in Iran found that the majority of malaria cases were male [16], while a study conducted by Okwa et al. in Nigeria showed that women were more likely to have malaria than males [17]. Women are expected to have better immunity to malaria and other types of parasitic diseases than men, and this has been attributed to genetic or hormonal factors [17,18]. The present study found that while the majority of cases (over 95%) were Afghans, approximately 3% of all malaria cases were Iranian (Table 1). A large number of workers come from malaria-endemic countries, such as Afghanistan and Pakistan, and they transmit malaria cases to these areas. However, a study in Iran showed that according to nationality, the majority of malaria cases were Iranian [19]. The study also showed that the majority of malaria cases were workers. A study in China found that workers do not have enough money to pay for malaria treatment [20]. A similar study in Iran also showed that workers were more likely to have malaria than others [16]. In the present study, the age group of 20–24 years had the highest incidence of malaria. A similar study found that men and women over the age of 15 had the highest number of malaria cases [16]. The predominant species of malaria in this study was *P. vivax*, which is similar to the results of the study carried out by Hanafi-Bojd et al. in Southern Iran [19]. However, in a study by Asante and colleagues in Ghana, it was found that *Plasmodium falciparum* was the dominant species with more than 98% prevalence [21]. In the present study, it was found that most malaria cases were imported in terms of epidemiological classification, while a study in Southern Iran showed that the majority of malaria cases were indigenous [22]. The presence of workers from other countries, especially Afghanistan and Pakistan, seems to have increased the number of malaria cases. The results of the present study showed that, in general, most cases of malaria occurred in urban areas, which disagrees with the results of Moemenbellah et al., who stated that most cases of malaria were seen in rural areas [23]. The frequency trend and incidence of malaria in one hundred thousand people per month showed that most of the new malaria cases occurred in the months of July to October. The peak incidence was in August. A study conducted in Malaysia has shown that the peak incidence of malaria was in the months of May to August [24]. One study found that June had the highest number of cases [23]. A study by Zacarias et al. in Mozambique showed that malaria incidence correlated significantly with humidity and maximum temperature, so that the malaria incidence will increase with rising environmental temperature [25]. The results of our study showed that the majority of malaria cases were detected through passive monitoring, which was consistent with the results of Moemenbellah’s study [23]. The results of Cochran-Armitage Trend test in this study showed that this trend was not statistically significant despite the mild upward trend in malaria incidence in the south of Fars province. The highest incidences were in the years 2008, 2016, and 2017, respectively, and the lowest in 2013. The reasons for the increase in malaria cases can be related to: the increasing number of malaria discovery laboratory tests, increased rainfall in recent years in the area providing conditions for *Anopheles* growth and proliferation, favorable temperature and humidity, and the increasing number of refugees and persons displaced from neighboring countries. On the other hand, increased surveillance activities in the prevention and control of malaria in recent years have reduced the number of cases. Therefore, with the above reasons, it can be concluded that the increase in the number of malaria cases has been slightly upward in recent years but with no statistically significant difference.

One of the limitations of the present study was the lack of access to all patient demographic information.

## Conclusion

The results of this study showed that the trend of malaria in the south of Fars province was slightly ascending, which was not statistically significant. Increased surveillance activities to prevent and control malaria disease as well as the suitability of climatic conditions and entry of people from endemic areas into the south of Fars province in southern Iran will have an impact on this trend. Since most cases of the disease were imported, educating people, especially refugees and displaced persons, to seek health care and therapeutic support can be effective in reducing malaria incidence. Additionally, environmental healthy and clean residential areas, both in the city and in the countryside, would make it difficult for *Anopheles* to grow and reproduce, which can help reduce malaria. Periodic checkups and diagnostic tests for high-risk individuals, such as refugees and Afghan workers, can also be helpful in early diagnosis of malaria.
